# Generic amplification and next generation sequencing reveal Crimean-Congo hemorrhagic fever virus AP92-like strain and distinct tick phleboviruses in Anatolia, Turkey

**DOI:** 10.1186/s13071-017-2279-1

**Published:** 2017-07-14

**Authors:** Ender Dinçer, Annika Brinkmann, Olcay Hekimoğlu, Sabri Hacıoğlu, Katalin Földes, Zeynep Karapınar, Pelin Fatoş Polat, Bekir Oğuz, Özlem Orunç Kılınç, Peter Hagedorn, Nurdan Özer, Aykut Özkul, Andreas Nitsche, Koray Ergünay

**Affiliations:** 1 Mersin University, Advanced Technology Education, Research and Application Center, 33110 Mersin, Turkey; 2 Robert Koch Institute; Center for Biological Threats and Special Pathogens 1 (ZBS-1), 13353 Berlin, Germany; 3 Faculty of Science, Department of Biology, Division of Ecology, Hacettepe University, 06800 Ankara, Turkey; 4 Faculty of Veterinary Medicine, Department of Virology, Ankara University, 06110 Ankara, Turkey; 5 Faculty of Veterinary Medicine, Department of Virology, Yuzuncu Yil University, 65080 Van, Turkey; 6 Faculty of Veterinary Medicine, Department of Internal Medicine, Harran University, 63200, Sanlıurfa, Turkey; 7 Yuzuncu Yil University, Ozalp Vocational School, 65080 Van, Turkey; 8 Faculty of Medicine, Department of Medical Microbiology, Virology Unit, Hacettepe University, 06100 Ankara, Turkey

**Keywords:** Tick, Crimean-Congo hemorrhagic fever virus, *Phlebovirus*, AP92, *Nairovirus*, Next generation sequencing, Surveillance, Anatolia, Turkey

## Abstract

**Background:**

Ticks are involved with the transmission of several viruses with significant health impact. As incidences of tick-borne viral infections are rising, several novel and divergent tick- associated viruses have recently been documented to exist and circulate worldwide. This study was performed as a cross-sectional screening for all major tick-borne viruses in several regions in Turkey. Next generation sequencing (NGS) was employed for virus genome characterization. Ticks were collected at 43 locations in 14 provinces across the Aegean, Thrace, Mediterranean, Black Sea, central, southern and eastern regions of Anatolia during 2014–2016. Following morphological identification, ticks were pooled and analysed via generic nucleic acid amplification of the viruses belonging to the genera *Flavivirus*, *Nairovirus* and *Phlebovirus* of the families *Flaviviridae* and *Bunyaviridae*, followed by sequencing and NGS in selected specimens.

**Results:**

A total of 814 specimens, comprising 13 tick species, were collected and evaluated in 187 pools. Nairovirus and phlebovirus assays were positive in 6 (3.2%) and 48 (25.6%) pools. All nairovirus sequences were closely-related to the Crimean-Congo hemorrhagic fever virus (CCHFV) strain AP92 and formed a phylogenetically distinct cluster among related strains. Major portions of the CCHFV genomic segments were obtained via NGS. Phlebovirus sequencing revealed several tick-associated virus clades, including previously-characterized Antigone, Lesvos, KarMa and Bole tick viruses, as well as a novel clade. A wider host range for tick-associated virus strains has been observed. NGS provided near-complete sequences of the L genomic segments of Antigone and KarMa clades, as well as Antigone partial S segment. Co- infections of CCHFV and KarMa or novel phlebovirus clades were detected in 2.1% of the specimens.

**Conclusions:**

Widespread circulation of various tick-associated phlebovirus clades were documented for the first time in Anatolia. Genomes of CCHFV AP92 strains were identified in previously unexplored locations. NGS provided the most detailed genomic characterization of the Antigone and KarMa viruses to date. The epidemiological and health-related consequences must be elucidated.

**Electronic supplementary material:**

The online version of this article (doi:10.1186/s13071-017-2279-1) contains supplementary material, which is available to authorized users.

## Background

Ticks are closely-associated with the epidemiology of several microbial pathogens with significant human and animal health impact [1]. They have been implicated as arthopod vectors of many viral, bacterial, and protozoal agent and their long life-cycle, expansive range and ability to feed on a wide array of vertebrates immensely contribute to their potential for disseminating infectious agents to susceptible hosts [2]. Classified in the class Arachnida, subclass Acari, the currently-described tick species have been included in the families Argasidae (argasid or soft ticks), Ixodidae (ixodid, or hard ticks) and Nuttalliellidae [3]. Argasid and ixodid ticks can transmit a surprisingly-broad range of viruses, bacteria and protozoans, surpassing most arthropods in terms of vector potential [4].

Many tick-borne viral pathogens, causing severe morbidity and mortality, are classified in the families *Flaviviridae* or *Bunyaviridae*, such as notable human pathogens tick-borne encephalitis virus (TBEV), Crimean-Congo hemorrhagic fever virus (CCHFV), Kyasanur forest disease virus, Powassan virus, Alkhurma virus, as well as livestock pathogens African swine fever virus, Nairobi sheep disease virus, and louping ill virus [5–7]. Sharing the common flavivirus structure and canonical genome organization, tick-borne flaviviruses have enveloped virions with a single-stranded RNA genome in positive orientation [8]. Viral genome possesses a single open reading frame (ORF) that encodes the viral polyprotein, which requires cleavage by viral and host proteases to form the mature structural (C, preM and E), and nonstructural (NS1, NS2A, NS2B, NS3, NS4A, NS4B and NS5) proteins [8]. Bunyaviruses are a diverse family of viruses with more than 400 distinct members grouped in five genera, where the major tick-borne species are classified in the genera *Nairovirus* and *Phlebovirus* [9]. All bunyaviruses are enveloped viruses with a tripartite, single-stranded RNA genome. Viral nucleocapsid protein (N), envelope glycoproteins (Gn and Gc), and RNA-dependent RNA polymerase are encoded in negative or ambisense orientation, from small (S), medium (M), and large (L) genome segments, respectively [9]. The lengths of the genomic segments vary among the genera, with the total genome lengths of approximately 11–19 kb. They also demonstrate genus-specific consensus sequences at the terminal ends of genome segments [10]. The vectors of these tick-borne infections mostly comprise ixodid ticks, classified in the genera *Ixodes*, *Haemaphysalis*, *Hyalomma*, *Amblyomma*, *Dermacentor*, *Rhipicephalus* and *Boophilus* [11].

Current evidence suggests a global increase in the incidence of tick-borne diseases [2]. This is not only due to an elevated frequency of endemic infections enhanced diagnosis in affected regions and expansion of tick activity zones associated with climatic factors, but to the discovery and emergence of viruses associated with ticks, as well. Several novel tick-borne viruses have been characterized within the last decade. Arguably the most prominent is the severe fever with thrombocytopenia syndrome virus (SFTSV), identified following local outbreaks of a febrile disease with high mortality rates in several provinces of China during 2009–2011 [12]. SFTSV cases or exposure has also been documented in South Korea and Japan; and *Haemaphysalis longicornis* ticks were suggested as the primary transmission vector [13, 14]. Another novel virus, the Heartland virus (HRTV), vectored via *Amblyomma americanum* ticks, has been characterized in human cases of febrile disease in the USA [15, 16]. SFTSV and HRTV are closely-related phleboviruses, and tentatively classified as a new group of tick-borne phleboviruses [17, 18]. The recent years have also witnessed the worldwide emergence of several novel tick-associated phleboviruses with unexplored human or animal health risks. Facilitated with the dissemination of next generation sequencing (NGS) technologies and application of viral metagenomics, sequences of novel strains, genetically-related to tick-borne group of phleboviruses have been characterized, from a wide range of geographical locations including the American continent [19], Africa [20, 21], Asia [22, 23], Australia [24] and Europe, around the Mediterranean region [25–27]. These findings clearly demonstrate the circulation of a spectrum of divergent strains in various parts of the globe and require investigation of the epidemiology as well as potential public health threats.

Located in Asia Minor and Eastern Thrace region of the Balkan Peninsula, Turkey covers a transboundary temperate climate zone between Asia and Europe. Turkey provides suitable habitats for several tick species due to the variety of ecological and climatic conditions observed throughout Anatolia [28]. Several tick-borne infections have been documented in Turkey, resulting in significant economic burden and pose major public health threats [29]. The most prominent tick-borne viral infection in Turkey is CCHF, which has emerged in 2002 and spread throughout Anatolia, with a total of 9787 cases and a mortality rate of 4.79% reported during 2002–2015 [30]. Other than CCHFV, very limited data on the presence or epidemiology of tick- borne viruses is currently available [29, 31]. This study was undertaken to perform a cross- sectional screening for all major tick-borne flaviviruses and bunyaviruses via generic PCR in several regions in Turkey, and characterize the identified strains via direct NGS on tick specimens.

## Methods

### Study area, specimen collection and identification

**Fig. 1:**
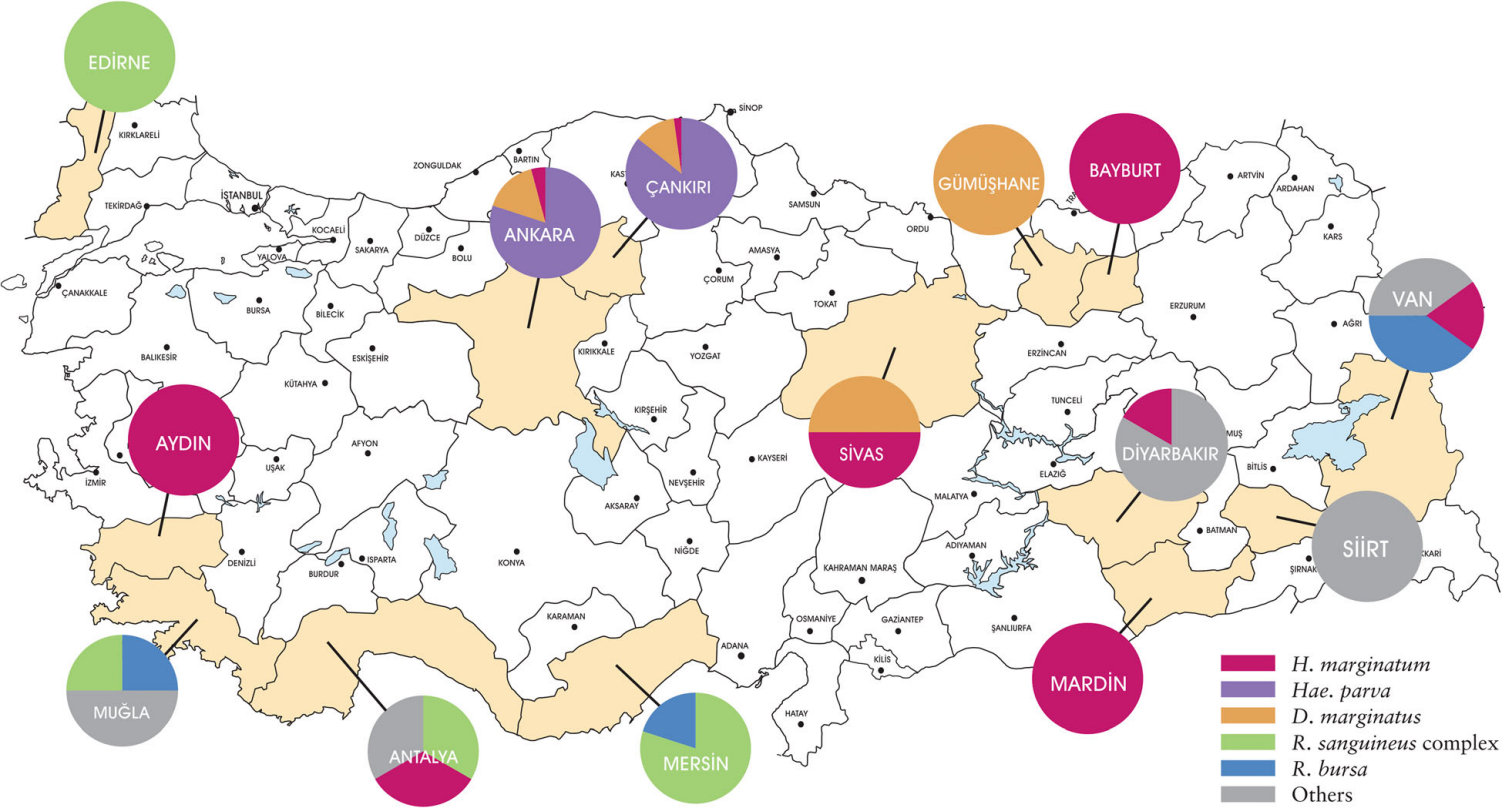
Illustrative map of sampling locations and distribution of the common tick species in the study

Tick sampling was carried out at 43 locations in Edirne (north-western Anatolia, Thrace region), Aydin and Mugla (western Anatolia, Aegean region), Ankara and Cankiri (central Anatolia), Sivas and Van (eastern Anatolia), Bayburt and Gumushane (northern Anatolia, Black Sea region), Antalya and Mersin (southern Anatolia, Mediterranean region), Diyarbakir, Mardin and Siirt (south-eastern Anatolia) provinces; from April to October during three consecutive years (2014–2016) (Fig. 1). At each site, flagging was performed to collect questing ticks and ticks infesting domesticated animals, mainly cattle (*Bos taurus*), sheep (*Ovis aries*), goats (*Capra aegagrus hircus*) and dogs (*Canis familiaris*) were collected at animal shelters. The specimens were kept alive in separate vials, transferred to the laboratory and identified morphologically to the species level using appropriate taxonomic keys [32–36]. Subsequently, ticks were pooled according to collection site, species and developmental stage up to a maximum of 20 individuals per pool and stored at -80 °C.

### Specimen processing, nucleic acid purification and cDNA synthesis

Individual and pooled ticks with up to 5 individuals were subjected to homogenization using the SpeedMill PLUS homogenizator (Analytikjena, Jena, Germany) and subsequently, total RNA extraction via BlackPREP tick DNA/RNA kit (Analytikjena, Jena, Germany) according to the manufacturer’s instructions. Tick pools with more than 5 individuals were homogenized by vortexing with tungsten carbide or stainless steel beads (Qiagen, Hilden, Germany) in 500–700 μl of Eagle’s minimal essential medium, supplemented with 5% foetal bovine serum and 1% L-glutamine. They were clarified by centrifugation at 1430× *g* for 4 min, aliquoted and stored at -80 °C. Nucleic acids purification was carried out in one aliquot via High Pure Viral Nucleic Acid Kit (Roche Diagnostics, Mannheim, Germany). All specimens were subsequently subjected to complementary DNA synthesis with random hexamers, using the RevertAid First Strand cDNA Synthesis Kit (Thermo Fisher Scientific, Hennigsdorf, Germany), performed according to the manufacturers’ guidelines.

### Virus screening via generic PCR

A spectrum of assays for the detection of viruses belonging to the genera *Nairovirus*, *Flavivirus* and *Phlebovirus* of the families *Flaviviridae* and *Bunyaviridae*, were employed for the screening of individual or pooled ticks. Generic *Nairovirus* screening was carried out via a previously published single round PCR protocol with primers targeting the central motif A of the viral polymerase (on the genomic segment L) [37]. The assay could amplify 14 representative strains of all serogroups in the genus *Nairovirus*, including CCHFV and Nairobi sheep disease virus. Previously quantitated human plasma from CCHFV-infected individuals were employed for assay optimization [38].

A nested PCR assay with degenerated primers targeting the NS5 conserved region was used for the flavivirus screening in ticks. The assay provides a sensitive amplification of all major tick and mosquito-borne pathogenic flaviviruses including West Nile virus (WNV), dengue virus, yellow fever virus, TBEV, Murray Valley encephalitis virus, Saint Louis encephalitis virus and Usutu virus [39]. Assay optimization was accomplished via WNV NY99–4132 isolate, propagated on African green monkey (Vero) cells (ATCC-CCL81) for mosquito- borne, and TBEV strain Hypr cDNA, obtained from the European Virus Archive (http://www.european-virus-archive.com) for tick-borne strains.

Phlebovirus screening was performed using two PCR assays optimized for the amplification of tick-borne phleboviruses [20]. The binding region of the primer sets target the well-conserved viral polymerase functional motifs premotif A and motif B [40], to facilitate detection of known or novel virus strains. The assays were reported to have detection limits of 102–103 tissue culture infectious dose (TCID50) equivalents of various tick-borne phleboviruses, following RNA extraction from culture supernatants [20]. Toscana virus isolate ISS.Phl.3, propagated on Vero cells and can be robustly detected via these primer sets, was used for optimization.

Amplified products of the screening assays were visualized in a ChemiDoc XRS+ imaging system (Bio-Rad Laboratories, Munich, Germany) via ethidium bromide staining after electrophoresis in 1.3–1.7% agarose gels. Extreme care was taken to prevent carry-over contamination, and extraction, pre- and post-PCR steps were performed in spatially-separated areas.

### Sequencing and phylogenetic analysis

Detectable PCR products of the screening assays were cleaned up using PureLink PCR Purification Kit (Thermo Fisher Scientific, Hennigsdorf, Germany) and characterized via sequencing in an ABI PRISM 3500xL Dx Genetic Analyzer (Thermo Fisher Scientific), using forward-reverse primers of the relevant assay and BigDye Terminator v3.1 Cycle Sequencing Kit (Thermo Fisher Scientific). Obtained sequences were handled using CLC Main Workbench v7.8.1 (CLCBio, Aarhus, Denmark), Bioedit v.7.0.9.0 and MEGA v.6.06 softwares [41, 42].

BLASTn, BLASTn optimized for highly similar sequences (MEGABLAST) and BLASTp algorithms were employed for nucleotide similarity searches in the public databases, implemented within the National Center for Biotechnology Information website (https://blast.ncbi.nlm.nih.gov/Blast.cgi) [43]. Nucleotide and putative amino acid alignments were generated via the CLUSTAL W program, implemented within the Bioedit software [44]. Pairwise sequence comparisons were carried out via MEGA and CLC softwares. The appropriate model for the phylogenetic and molecular evolutionary analyses was determined using the Find best DNA/protein-substitution model tools built into MEGA software. Maximum likelihood trees were constructed using the Tamura-Nei and Jones-Taylor-Thornton models, based on the nucleotide and amino acid sequences, respectively. The reliability of the inferred trees was evaluated by bootstrap analysis of 1000 pseudoreplicates. Conserved protein domain and motif searches were performed using the Web CD - search tool (https://www.ncbi.nlm.nih.gov/Structure/cdd/wrpsb.cgi) and MOTIF Search (http://www.genome.jp/tools/motif/) in the PFAM database [45, 46].

### Next generation sequencing (NGS) and data analysis

Aliquoted RNA of the selected tick specimens were reverse transcribed using random hexamers into double-stranded cDNA via SuperScript IV Reverse Transcriptase (Thermo Fisher Scientific, Hennigsdorf, Germany) and NEBNext mRNA Second Strand Synthesis Module (New England Biolabs, Frankfurt am Main, Germany). Agencourt AMPure XP Reagent (Beckman Coulter Biosciences, Krefeld, Germany) and Agilent 2100 Bioanalyzer (Agilent Technologies, Waldbronn, Germany) were used for the cleanup, yield and size distribution determination.

Fragmentation, adaptor ligation and amplification steps were performed via the NexteraXT DNA Library Preparation Kit (Illumina Inc., San Diego, CA, USA), as indicated by the manufacturer. The sequencing run was performed in one lane of the Illumina HiSeq 1500 (Illumina Inc.) in the high output mode.

The raw sequencing data was de-multiplexed and extracted in fastq format. Trimmomatic and Bowtie2 v2.2.9 softwares were employed for trimming for quality and length, removal of adaptor and background mapping [47, 48]. Acquired reads were aligned to the RefSeq viral nucleotide and protein genome database using MALT (MEGAN alignment tool, v0.3.8) and DIAMOND v0.7.1 tools [49, 50]. De novo assembly of the virus genomic segments were carried out using Geneious software v9.1 (Biomatters Ltd., Auckland, New Zealand).

## Results

A total of 814 specimens, comprising 13 tick species, were collected (Additional file 1: Table S1). The specimens include 299 ticks (36.8%) from central Anatolian provinces, 255 (31.3%) from Mediterranean provinces, 191 (23.4%) from Aegean/Thrace provinces, 32 (3.9%) from eastern Anatolian provinces, 21 (2.6%) from south-eastern Anatolian provinces and 16 (1.9%) from Black Sea provinces. The most abundant tick species was *Riphicephalus sanguineus* complex (*n* = 263; 32.3%), followed by *Haemaphysalis parva* (*n* = 254; 31.2%), *Riphicephalus bursa* (*n* = 96; 11.8%) and others (Additional file 1: Table S1). The distribution of frequently- observed tick species according to the sampling province is provided in Fig. 1. Tick specimens were grouped into 187 pools, that comprise 66 pools (35.2%) from Mediterranean provinces, 49 (26.2%) from central Anatolian provinces, 27 (14.4%) from Aegean/Thrace provinces, 25 (13.3%) from eastern Anatolian provinces, 18 (9.6%) from south-eastern Anatolian provinces and 2 (1%) from Black Sea provinces (Additional file 1: Table S1**)**.

### Virus screening

A total of 50 pools (50/187, 26.7%) were positive in the screening assays, originating from central Anatolian (Cankiri: 1 pool), eastern Anatolian (Van: 12 pools), Black Sea (Bayburt: 1 pool), south-eastern Anatolian (Diyarbakir: 2 pools, Siirt: 1 pool), Mediterranean (Mersin: 28 pools) and Aegean (Mugla: 5 pools) provinces (Table 1). Generic nairovirus PCR was positive in 6 pools, collected from 3 provinces in eastern Anatolia, Black Sea and Mediterranean regions (Table 1). Positive pools comprised *R. bursa* (3/6) *R. sanguineus* complex (2/6) and *Hyalomma marginatum* (1/6) specimens. Generic tick phlebovirus PCR revealed positive results in 48 pools, collected from 6 provinces in eastern, south-eastern and central Anatolia, Aegean and Mediterranean regions (Table 1). Phlebovirus-positive pools comprised *R. sanguineus* complex (31/48), *R. bursa* (8/48), *H. marginatum* (4/48), *Hyalomma aegyptium* (3/48), *Hyalomma excavatum* (1/48) and *Haemaphysalis parva* (1/48) specimens. In 4 pools with *R. bursa* (3/4) *R. sanguineus* complex (1/4) specimens, nairovirus and phlebovirus co-infections were identified, with both assays providing positive results (Table 1). All specimens were negative in the generic flavivirus PCR. The contents of all reactive pools are provided in Tables 2 and 3.

**Table 1:** Screening results according to the sampling region

Province	Tick pools		Virus detected	Co-infection	
	Number	Positive *n* (%)	CCHFV	Phlebovirus	
Ankara	12	0	–	–	–
Antalya	3	0	–	–	–
Aydin	1	0	–	–	–
Bayburt	1	1 (100)	1	0	0
Cankiri	37	1 (2.7)	0	1	0
Diyarbakir	13	2 (15.4)	0	2	0
Edirne	1	0	–	–	–
Gumushane	1	0	–	–	–
Mardin	1	0	–	–	–
Mersin	63	28 (44.4)	2	27	1
Mugla	25	5 (20.0)	0	5	0
Siirt	4	1 (25.0)	0	1	0
Sivas	2	0	–	–	–
Van	23	12 (52.2)	3	12	3
Total	187	50 (26.7)	6 (3.2)	48 (25.6)	4 (2.1)

**Table 2:** Features of the tick pools characterized via amplicon sequencing

Code		Province	Species	Pool composition	Host collection method	Virus detected	GenBank accession number
1	KM6	Mersin	*R. sanguineus* complex	1♀, 1♂	Dog	CCHFV	KY963543 (CCHFV)
2	KM3	Mersin	*R. sanguineus* complex	4♀, 6♂	Dog	Tick *Phlebovirus* (novel lineage) - CCHFV	KY965971 (*Phlebovirus*), KY963544 (CCHFV)
3	ET35	Van	*R. bursa*	1♂	Sheep	Tick *Phlebovirus* (KarMa lineage) -CCHFV	KY979161 (*Phlebovirus*), KY963542 (CCHFV)
4	ET36	Van	*R. bursa*	1♀	Sheep	Tick *Phlebovirus* (KarMa lineage) -CCHFV	KY966010 (*Phlebovirus*), KY963540 (CCHFV)
5	ET37	Van	*R. bursa*	1♀	Goat	Tick *Phlebovirus* (KarMa lineage) -CCHFV	KY966002 (*Phlebovirus*), KY963541 (CCHFV)
6	KM2	Mersin	*R. sanguineus* complex	2♀, 1 nymph	Dog	Tick *Phlebovirus* (novel lineage)	KY965970
7	KM11	Mersin	*R. sanguineus* complex	2♀, 3♂	Dog	Tick *Phlebovirus* (novel lineage)	KY965972
8	KM12	Mersin	*R. sanguineus* complex	3♀	Dog	Tick *Phlebovirus* (novel lineage)	KY965992
9	KM15	Mersin	*R. sanguineus* complex	1♀	Dog	Tick *Phlebovirus* (novel lineage)	KY965973
10	KM17	Mersin	*R. sanguineus* complex	9♀, 2♂	Dog	Tick *Phlebovirus* (novel lineage)	KY965974
11	KM19	Mersin	*R. sanguineus* complex	1♀, 1♂	Dog	Tick *Phlebovirus* (novel lineage)	KY965993
12	KM21	Mersin	*R. sanguineus* complex	4♀	Dog	Tick *Phlebovirus* (novel lineage)	KY965975
13	KM22	Mersin	*R. sanguineus* complex	2♀, 1♂	Dog	Tick *Phlebovirus* (novel lineage)	KY965976
14	KM23	Mersin	*R. sanguineus* complex	3♀, 2♂	Dog	Tick *Phlebovirus* (novel lineage)	KY965977
15	KM24	Mersin	*R. sanguineus* complex	2♀, 3♂	Dog	Tick *Phlebovirus* (novel lineage)	KY965995
16	KM25	Mersin	*R. sanguineus* complex	3♀, 2♂	Dog	Tick *Phlebovirus* (novel lineage)	KY965978
17	KM26	Mersin	*R. sanguineus* complex	4♀, 4♂	Dog	Tick *Phlebovirus* (novel lineage)	KY965979
18	KM27	Mersin	*R. sanguineus* complex	1♀, 1♂	Dog	Tick *Phlebovirus* (novel lineage)	KY965980
19	KM29	Mersin	*R. sanguineus* complex	1♀	Dog	Tick *Phlebovirus* (novel lineage)	KY965981
20	KM30	Mersin	*R. sanguineus* complex	5♀, 1♂	Dog	Tick *Phlebovirus* (novel lineage)	KY965982
21	KM31	Mersin	*R. sanguineus* complex	7♀, 1♂	Dog	Tick *Phlebovirus* (novel lineage)	KY965994
22	KM33	Mersin	*R. sanguineus* complex	1♂	Dog	Tick *Phlebovirus* (novel lineage)	KY965983
23	KM35	Mersin	*R. sanguineus* complex	1♀, 2♂	Dog	Tick *Phlebovirus* (novel lineage)	KY965984
24	KM41	Mersin	*R. sanguineus* complex	1♀	Dog	Tick *Phlebovirus* (novel lineage)	KY965998
25	KM49	Mersin	*R. sanguineus* complex	2♀, 2♂	Dog	Tick *Phlebovirus* (novel lineage)	KY965985
26	KM50	Mersin	*R. sanguineus* complex	2♀, 2♂	Dog	Tick *Phlebovirus* (novel lineage)	KY965986
27	KM51	Mersin	*R. sanguineus* complex	1♀, 5 nymphs	Dog	Tick *Phlebovirus* (novel lineage)	KY965987
28	KM55	Mersin	*R. sanguineus* complex	2♀	Dog	Tick *Phlebovirus* (novel lineage)	KY965988
29	KM59	Mersin	*R. sanguineus* complex	2♀	Dog	Tick *Phlebovirus* (novel lineage)	KY965989
30	KM60	Mersin	*R. sanguineus* complex	2♀, 1♂	Dog	Tick *Phlebovirus* (novel lineage)	KY965990
31	KM36	Mersin	*R. sanguineus* complex	3♂, 1♀	Dog	Tick *Phlebovirus* (Lesvos lineage)	KY965991
32	CA16	Cankiri	*Hae. parva*	17♀	Goat	Tick *Phlebovirus* (Lesvos lineage)	KY965997
33	MU43	Mugla	*R. sanguineus* complex	2♀	Dog	Tick *Phlebovirus* (novel lineage)	KY965996
34	ET2	Van	*R. bursa*	1♀	Cattle	Tick *Phlebovirus* (KarMa lineage)	KY966006
35	ET20	Van	*R. sanguineus* complex	1♂	Cattle	Tick *Phlebovirus* (Antigone lineage)	KY966007
36	ET11	Van	*H. marginatum*	1♂	Sheep	Tick *Phlebovirus* (Bole lineage)	KY966005
37	ET25	Van	*H. aegyptium*	1♀	Sheep	Tick *Phlebovirus* (Bole lineage)	KY979160
38	ET26	Van	*H. aegyptium*	1♀	Goat	Tick *Phlebovirus* (Bole lineage)	KY965999
39	ET29	Van	*H. aegyptium*	1♂	Sheep	Tick *Phlebovirus* (Bole lineage)	KY966003
40	ET31	Van	*R. bursa*	1♀	Goat	Tick *Phlebovirus* (KarMa lineage)	KY966011
41	ET32	Van	*R. bursa*	1♀	Goat	Tick *Phlebovirus* (KarMa lineage)	KY966008
42	ET33	Van	*R. bursa*	1♀	Sheep	Tick *Phlebovirus* (KarMa lineage)	KY966009
43	ET6	Diyarbakir	*H. excavatum*	1♂	Cattle	Tick *Phlebovirus* (Bole lineage)	KY966001
44	ET14	Diyarbakir	*H. marginatum*	1♂	Sheep	Tick *Phlebovirus* (Bole lineage)	KY966000
45	ET7	Siirt	*H. marginatum*	1♂	Sheep	Tick *Phlebovirus* (Bole lineage)	KY966004

**Table 3:** Features of the tick pools characterized via next generation sequencing

Code		Province	Species and composition	Host collection method	Virus detected	Genomic target	Metagenome viral reads	Length	GenBank accession number
1	MG22	Mugla	*R. sanguineus* complex (1♀)	Flagging	Tick Phlebovirus (Antigone clade)	L segment	4000	6539	KY979165
						S segment	155	440	KY984485
2	MG31	Mugla	*R. bursa* (6♀, 6♂)	Flagging	Tick Phlebovirus (Antigone clade)	L segment	343	6539	KY979166
3	MG36	Mugla	*R. sanguineus* complex (1♂)	Flagging	Tick Phlebovirus (Antigone clade)	L segment	1100	6374	KY979167
4	MG49	Mugla	*H. marginatum* (5♀)	Cattle	Tick Phlebovirus (KarMa clade)	L segment	544	6363	KY979168
5	MG48	Bayburt	*H. marginatum* (9♀, 4♂)	Cattle	CCHFV (AP92-like)	L segment	2863	11,988	KY979162
						M segment	318	3852	KY979163
						S segment	228	1484	KY979164

### Analysis and characterization of nairovirus sequences

**Fig. 2:**
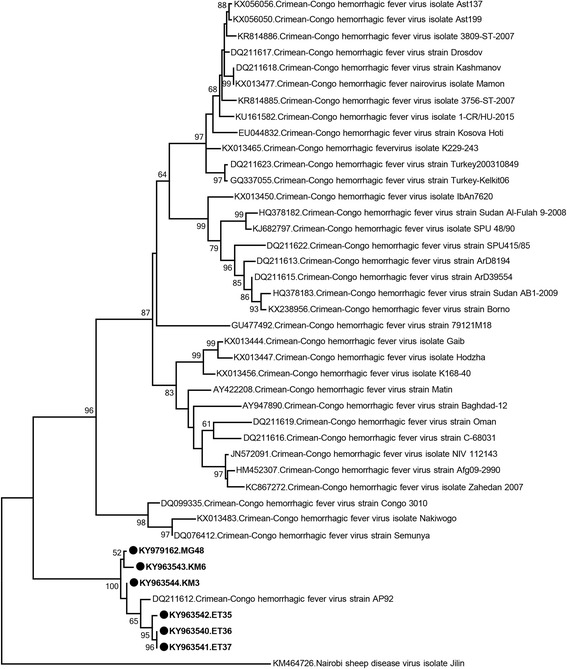
Maximum likelihood analysis of the partial Crimean-Congo hemorrhagic fever virus L segment sequences (445 nucleotides). The sequences characterized in this study are given in bold and indicated with a black circle, the GenBank accession number and pool code. Global virus strains are indicated by the GenBank accession number, virus and strain/isolate name. Bootstrap values higher than 50 are shown. Nairobi sheep disease virus isolate Jilin was included as the outgroup

In 6 generic nairovirus-positive specimens, sequences of 446–463 nucleotides were obtained. They demonstrated 0.9–3.0% variation and BLASTn and MEGABLAST searches revealed the most closely-related entry to be the CCHFV AP92 strain (E-value = 0.0). The sequences aligned to the 6705–7168 nucleotides on the L segment of the AP92 genome (GenBank accession number: DQ211612) and displayed 2.3–2.5% diversity from this strain. In the maximum likelihood analysis, the sequences formed a separate monophyletic cluster with the AP92 strain, distinct from other CCHFV clades, supported by high bootstrap values (Fig. 2). CCHFV sequences were detected in *H. marginatum*, *R. sanguineus* complex and *R. bursa*, collected from Mersin, Van and Bayburt provinces of Mediterranean and north-eastern Anatolia, in female and male individuals as well as pooled specimens (Tables 2, 3).

### Analysis and characterization of phlebovirus sequences

**Fig. 3:**
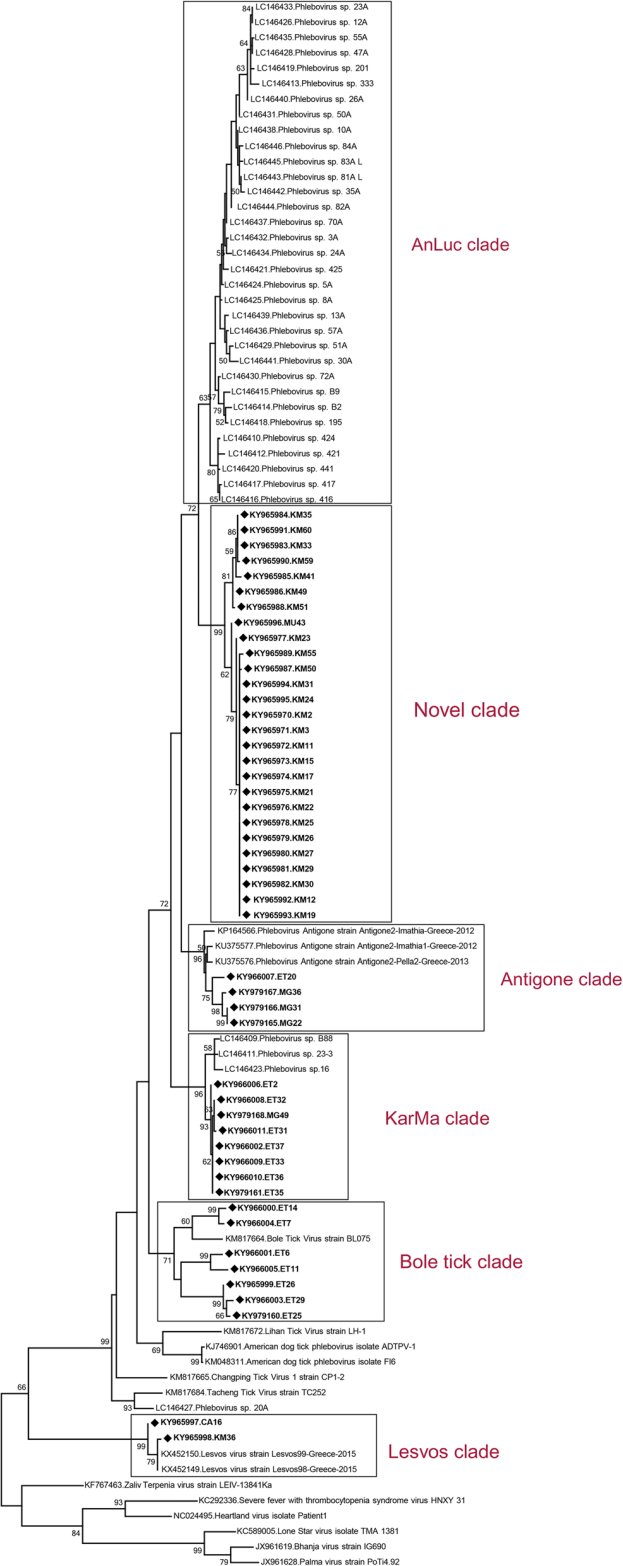
Maximum likelihood analysis of the partial tick-associated phlebovirus L segment sequences (258 nucleotides). The sequences characterized in this study are given in bold and indicated with a black diamond, the GenBank accession number and pool code. Global virus strains are indicated by the GenBank accession number, virus and strain/isolate name. Bootstrap values higher than 50 are shown

Sequencing of the generic phlebovirus amplicons provided sequences of 445–539 nucleotides with 53–100% similarity in pairwise comparisons. BLASTn and MEGABLAST analyses displayed similarities of varying degrees to several phlebovirus sequences, recently identified in Greece [25, 26], Portugal [27], USA [19] and China [23]. Maximum likelihood analysis revealed that the sequences formed distinct clades with previously-characterized tick phlebovirus sequences (Fig. 3). Four and 2 of the sequences were grouped with Antigone and Lesvos strains, identified in Greece, whereas another 8 sequences formed a distinct group with the sequences belonging in the KarMa clade, characterized in Portugal. Moreover, seven sequences grouped with the Bole tick virus, identified in China. However, the majority of the characterized sequences formed a distinct clade, which shares a common ancestor with the AnLuc lineage from Portugal (Fig. 3). Low nucleotide diversity rates were noted between sequences within the described clades; with 95.3% similarity for the Lesvos virus, 94–98.6% for KarMa virus, 90.1–99.4% for Antigone virus, 77–98% for Bole tick virus and 92.2–100% for the novel clade.

Phleboviruses Antigone, KarMa, Bole and the novel clade were detected in pools, as well as individual female and male ticks (Tables 2, 3). Association of the infection with sex could not be precisely determined for the Lesvos clade, as the sequences were detected in pools with female individuals and in mixed (Table 2). The Antigone and KarMa clades demonstrated a ubiquitous distribution, as these sequences could be detected in Van and Mugla provinces in eastern and western (Aegean) Anatolia. Similarly, Lesvos sequences were present in pools from Cankiri and Mersin provinces from central and southern (Mediterranean) Anatolia. Sequences belonging in the novel clade also originated from southern and western Anatolia. However, all specimens within the Bole clade were collected from Diyarbakir, Siirt and Van provinces of eastern and south-eastern Anatolia. This clade was also found exclusively in ticks of the genus *Hyalomma*. Likewise, *Rhipicephalus* spp. ticks were observed to harbour the Antigone clade and sequences of the novel clade was detected in *R. sanguineus* complex ticks (Tables 2, 3). Phlebovirus sequences could be detected in questing ticks as well as in ticks collected from vertebrate hosts.

### NGS findings on phlebovirus-positive specimens

**Fig. 4:**
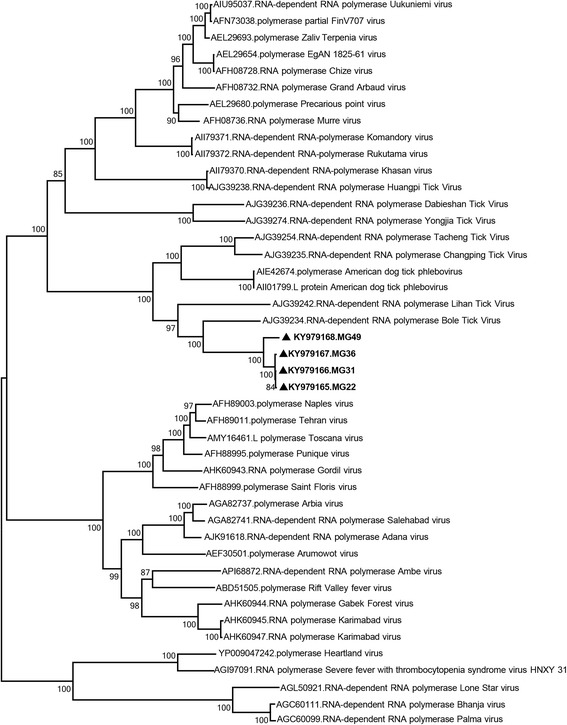
Maximum likelihood analysis of the putative, near-complete RNA-dependent RNA polymerase protein (2295 amino acids) of phleboviruses. The sequences characterized in this study are given in bold and indicated with a black triangle, the GenBank accession number and pool code. Global virus strains are indicated by the GenBank accession number, virus and strain/isolate name

Four specimens, initially positive via the phlebovirus screening assays were evaluated via NGS and sequences of 6363–6539 nucleotides were assembled (Table 3). They demonstrated 80–99% and 87.6–99.5% similarity at the nucleotide and putative amino acid levels, respectively (Additional file 2: Table S2), and were related to the L segment of Antigone virus and other tick- borne phleboviruses in BLASTn and MEGABLAST searches. The sequences were identified to represent the Antigone (specimens MG22, MG31 and MG36) and KarMa (MG49) clades via alignment and maximum likelihood analysis with the sequence data from the screening assays (Fig. 4).

In specimens MG22 and MG31, sequences of 6539 nucleotides were characterized, that represents the near-complete L segment of the Antigone clade. The putative coding regions were located identically, which encompassed the nucleotides 36–6488 on the sequence that encoded a 2161 amino acid product. Though non-coding regions of 35 and 51 nucleotides could be obtained via NGS, the sequences are considered near-complete since the phlebovirus-specific terminal motifs could be identified partially and no additional experiment was performed to ensure the availability of the complete L segment. *In silico* functional analyses of the polyprotein revealed several conserved motifs including the Bunyavirus N-terminus endonuclease domain (nucleotides 53–124; PSSMID: 317,864, PFAM-ID: PF15518) [51]; RNA-dependent RNA- polymerase (nucleotides 647–877 and 886–1313; PSSM-ID: 282,102, PFAM-ID: PF04196) and a viral protein of unknown function (DUF3770, nucleotides 174–426; PSSM-ID: 289,377, PFAM-ID: PF12603).

In specimens MG36 and MG49, 6374 and 6363 nucleotide-long stretches within the coding region could be obtained, which corresponded to the 2124 and 2121 amino acids of the viral RNA-dependent RNA-polymerase. The maximum likelihood analysis based on the putative amino acid sequences revealed a well-supported separation of the Antigone and KarMa clades, sharing common ancestors with Bole and Lihan tick viruses (Fig. 4). Pairwise comparison of the nucleotide and putative amino acid sequences of the L segment of tick phleboviruses with related strains are provided in Additional file 2: Table S2.

Moreover, a relatively short stretch (of 440 nucleotides) from the Antigone phlebovirus S genomic segment could be characterized via direct NGS in specimen MG22 (Table 3). BLASTn search have identified the Bole tick virus strain BL075 to be the most-closely-related strain (E- value: 2e-13) and similarity rates of 63.6 and 55.4% on the nucleotide and amino acid levels, respectively, were noted. In the maximum likelihood tree, a common ancestor of the partial Antigone S segment sequence and Bole and Lihan tick virus group were observed (Additional file 3: Figure S1). Pairwise comparison of the Antigone clade S segment sequence with related strains are provided in Additional file 4: Table S3.

### NGS findings on nairovirus positive specimens

**Fig. 5:**
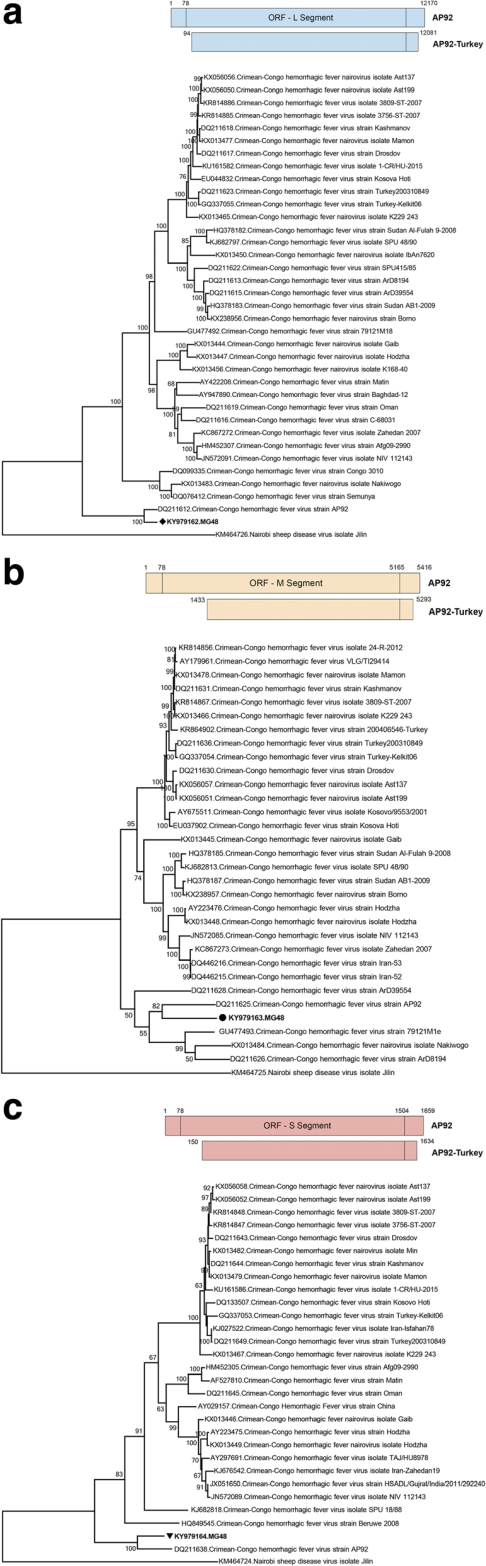
Schematic comparison and maximum likelihood analysis of the Crimean-Congo hemorrhagic fever virus L (**a**), M (**b**) and S (**c**) segments and coding/non-coding regions. The analysis is based on 13,125, 3928 and 1509 nucleotides for L, M and S segments, respectively. The characterized sequences are given in bold and indicated with symbols, the GenBank accession number and pool code. Global virus strains are indicated by the GenBank accession number, virus and strain/isolate name. Bootstrap values higher than 50 are provided. Nairobi sheep disease virus isolate Jilin was included as the outgroup

One specimen (MG48) that comprised *H. marginatum* specimens collected from Bayburt province of the Black Sea region was evaluated via direct NGS, following generic nairovirus PCR reactivity (Table 3). Sequences representing 3 genomic segments of the infecting nairovirus were assembled. BLASTn and MEGABLAST searches identified the CCHFV AP92 strain as the most closely-related nairovirus for all segments. Pairwise comparison of the data from each segment demonstrated that a significant portion of the coding regions and stretches of varying lengths from the 3′ non-coding ends could be characterized (Fig. 5). The strain is tentatively called as the AP92-Turkey and demonstrated 95.1, 76.2 and 90.5% nucleotide similarity of L, M and S segment sequences, respectively, with the prototype AP92 strain. Complete nucleotide and putative amino acid comparisons of viral genomic segments with various CCHFV lineages are provided in Additional files 5, 6 and 7: Tables S4-S6.

The obtained S segment sequence has further been aligned and analysed with previously- characterized AP92-like partial sequences from Turkey as well as several Balkan countries.

**Fig. 6:**
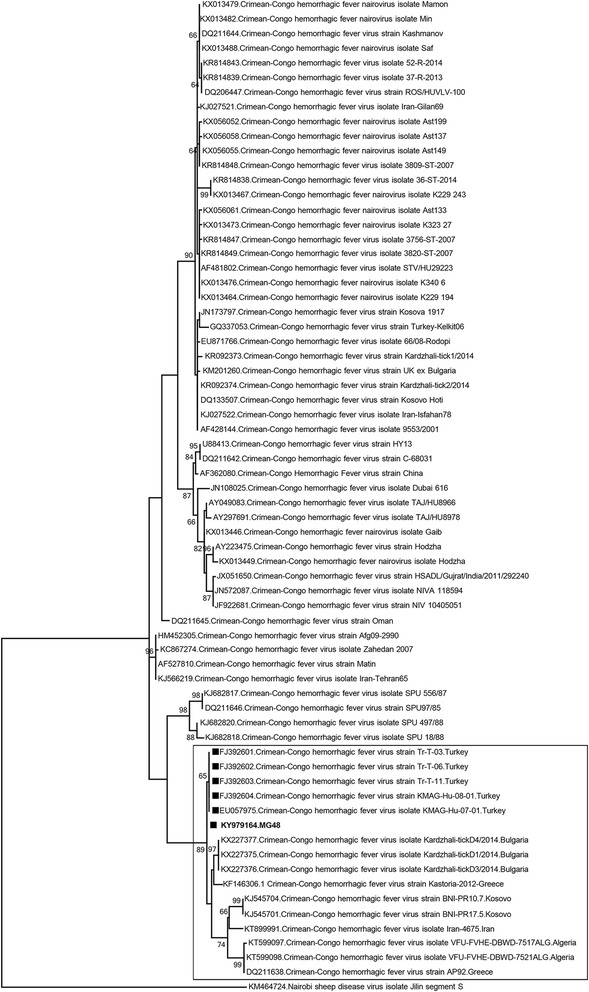
Maximum likelihood analysis of the partial Crimean-Congo hemorrhagic fever virus S segment sequences (174 nucleotides). The AP92 and related sequences are marked. Sequences obtained in Turkey are indicated with a black square and the GenBank accession number. The sequence characterized in this study is given in bold and with the pool code. Global virus strains are indicated by the GenBank accession number, virus and strain/isolate name. Bootstrap values higher than 60 are shown. Nairobi sheep disease virus isolate Jilin was included as the outgroup

Pairwise comparison of a 426-nucleotide partial sequence revealed 98.3–99.5% similarity with sequences previously-identified in ticks (GenBank: FJ392601– FJ392603) and humans (GenBank: EU057975 and FJ392604) in Thrace region of Turkey. These sequences from Turkey and AP92-related sequences characterized from Albania, Bulgaria, Iran, Greece and Kosovo formed distinct clusters in the maximum likelihood analysis (Fig. 6).

## Discussion

Vector surveillance provides an efficient tool for monitoring the introduction or circulation of emerging vector-borne pathogens into susceptible populations and provide an early warning system for predicting outbreaks [52]. Furthermore, they facilitate the accumulation of information on pathogen epidemiology, associations with vectors and potential impact on human/animal welfare. Turkey provides suitable ecological niches and the tick fauna that favor the introduction and establishment of a wide range of tick-borne viral agents, as exemplified by the emergence of CCHFV [28–30]. This study was performed to fill the current information gap on the circulation of tick-borne viral agents via a cross-sectional vector surveillance encompassing several geographically-separated regions of Turkey. The strategy of employing generic PCR assays for a broad screening of tick-borne viruses from various families/genera and subsequent NGS was adopted, making this study the most extensive tick-borne virus surveillance effort performed in Turkey to date. During nairovirus screening, we have detected CCHFV nucleic acids in 3.2% of the specimens, collected from Mediterranean, Black Sea and eastern Anatolian provinces. All strains were genetically-related to the CCHFV AP92 strain, isolated originally from northern Greece from *R. bursa* ticks infecting goats in 1975 [53]. The AP92 strain is divergent from all known CCHFV strains, forming a separate clade (often referred to as the Europe 2 lineage) [54, 55]. AP92 and related strains are known to circulate in several CCHFV-endemic regions such as in the Balkan Peninsula and Iran [53–58]. In Turkey, AP92-like sequences have previously been detected in *R. bursa* and *H. marginatum* ticks, collected from Edirne and Kirklarelis provinces of the Thrace region [59]. Moreover, cases presenting as a febrile form of CCHFV disease have been described from this region, as well [60, 61]. Our findings indicate a larger dissemination zone for AP92 in Turkey, including southern and eastern Anatolia. The AP92-related sequences have also been identified in *R. bursa* ticks from Greece and Kosovo [54, 57], in *R. sanguineus* complex from Bulgaria [55] and in *H. aegyptium* ticks from Algeria [56]. This widespread dissemination and infrequent detection in clinical disease have led to the assumption that AP92 and related strains may be causing a milder disease in affected individuals [54]. However, a recently-documented hemorrhagic fever case with fatal outcome in Iran indicates that severe infections similar to those induced by other CCHFV lineages occur [58]. Direct NGS performed on an infected tick pool enabled the near-complete genomic characterization of the Turkish.

AP92-related strain in this study, confirming the phylogenetic relations of various CCHFV lineages and AP92 (Fig. 5). Furthermore, the analysis of partial S segment sequences has revealed distinct clusters among AP92 strains, one of which constitutes all sequences identified in Turkey (Fig. 6). However, a direct geographical association is not evident and intramural divergence within AP92-like strains related to the vector, host or the collection site needs to be addressed in future efforts.

The screening for tick-borne phleboviruses revealed a wide-spread distribution and a detection rate of 23.5%, in 7 of the 14 provinces involved and from Aegean, Mediterranean, central, eastern and south-eastern Anatolia (Table 1). Furthermore, circulation of multiple and divergent strains have been observed, with 4 previously-identified clades as well as a novel one (Fig. 3, Table 2). Currently, 3 groups of tick-associated viruses have been recognized within the genus *Phlebovirus*; namely the SFTSV, the Bhanja group, and the Uukuniemi groups [17, 18]. However, several recently identified sequences, as well as those reported in this study, are genetically distinct from these groups (Fig. 3). This finding indicates that only a portion of the actual tick-associated phlebovirus spectrum could be identified so far. In order to designate overall genetic relatedness and to prevent misinterpretation, we have retained the previously used nomenclature in this report [19, 23, 25–27]. Among the detected clades, the Antigone and Lesvos viruses have been characterized in mainland Greece and Lesvos Island in *R. sanguineus* complex and *Hae. parva* species, respectively [25, 26]. Here, we have documented a wider host range for both strains via the detection of Antigone sequences in *R. bursa* and Lesvos sequences in *R. sanguineus* complex ticks (Tables 2, 3). KarMa is among the novel phleboviruses reported from southern Portugal, along with AnLuc and RiPar clades [27]. This clade has originally been detected in *R. sanguineus* complex and *H. marginatum* ticks. Infected ticks were identified as *R.bursa* and *H. marginatum* in this study, suggesting various *Rhiphicephaus* ticks to be susceptible. A wide-spread distribution of Antigone, Lesvos and KarMa clades in Anatolia is likely, since they could be detected in ticks from ecologically-diverse regions.

Another phlebovirus sequence group identified in this study has been named after Bole tick virus, characterized during a metagenomic survey of the field-collected *Hyalomma asiaticum* ticks in China [23]. These sequences demonstrated the highest intramural nucleotide variation (up to 23%) and phylogenetic distance among the clades detected in this study (Fig. 3); thus, may be representing a group of genetically related sequences. Interestingly, the sequences could only be detected in *Hyalomma* spp., collected from eastern, inland regions of Anatolia (Table 2). Lastly, we have characterized several phlebovirus sequences that shared a common ancestor with the AnLuc clade from Portugal in this study [27]. We suggest these sequences to constitute a novel clade among tick-associated phleboviruses. We have refrained from specifying a tentative name, until detailed genome characterization or virus isolation has been attained.

Sequences belonging in the novel clade could be detected in *R. sanguineus* complex ticks from southern and western Anatolia (Table 2). Consistent with the preliminary reports from Greece, Portugal and China [23, 25–27], the overall phlebovirus screening findings indicate that a spectrum of viral clades circulate in various geographical regions, in questing or host-associated ticks of both sexes.

Another interesting finding of this study is the identification of phlebovirus and CCHFV co-infections in 8% (4/50) of the specimens with detectable viral nucleic acids (Table 1). The KarMa and novel clades have been observed to participate in co-infections, identified in pooled as well as in individual female and male ticks from various regions (Table 2). These findings indicate that co-infections are not extremely rare and may be contributing to virus circulation dynamics. It remains to be explored whether genetic material exchange via recombination or re- assortment, as documented for several bunya and phleboviruses [62, 63], occurs within the tick host or phlebovirus infections affect vector competence of ticks for CCHFV. Despite previous serological findings suggesting rare occurrence of tick-borne encephalitis virus exposure [29, 31], no evidence of flavivirus circulation could be identified in this study. This is presumably due to the lack of *Ixodes* specimens in the study cohort (Additional file 1: Table S1).

The NGS performed on selected specimens with screening reactivity have resulted in the characterization of L and S segment sequences of the Antigone and KarMa clade phleboviruses, for which only limited data have been previously available. The near-complete L segment sequences have enabled a more robust analysis of the genetic distances among partially- characterized tick-borne phleboviruses, confirming the separation of these particular clades (Fig. 4). Furthermore, this approach has provided considerable genomic sequence data of the AP92- like CCHFV strain circulating in Anatolia. As they have been instrumental in generating the information on phlebovirus diversity [19, 20, 22, 23], NGS and similar high-throughput sequencing technologies will likely to be employed for the upcoming biosurveillance efforts as well.

An important issue that remains to be elucidated is whether these identified phleboviruses are pathogenic for humans or animals or not. Among the recently-described tick-borne phleboviruses, SFTSV and Heartland virus are documented human pathogens and SFTSV is a highly-pathogenic agent causing hemorrhagic fever with a case fatality rate as high as 50% [12–15, 64]. Moreover, Uukuniemi and Bhanja viruses, the representative strains of their groups, have been associated with human febrile diseases in specific cases [65–67]. However, the potential virulence of other tick-borne phleboviruses, including the Antigone, Lesvos and KarMa clades detected in this study, is not currently known and they constitute potential candidates for neglected agents of febrile diseases of unknown etiology. Given the serologically-proven human and animal exposure of Uukuniemi and Bhanja viruses [68–70], it is likely that these clades have already been in contact with various vertebrates via ticks in endemic regions, with unexplored consequences. The current lack of an isolated strain despite previous trials [25–27] hampers the serological investigation of past exposures. Studies are underway by our group to inoculate the infected tick specimens onto appropriate cell lines for a successful virus isolation and detailed characterization.

## Conclusion

We have documented the presence of multiple and divergent tick-associated phleboviruses throughout Anatolia, with four previously-identified as well as a novel clade. A wider host range for particular tick phlebovirus clades and a larger dissemination zone for the CCHFV AP92-like strains have been revealed. NGS has enabled the comprehensive genomic characterization of two phlebovirus clades and the CCHFV AP92-like strain in circulation.

## Additional files

Additional file 1: Table S1. Distribution of the tick specimens according to species, sex and collection province. (XLS 31 kb)

Additional file 2: Table S2. Pairwise comparison of the nucleotide and putative amino acid sequences of the L segment of tick phleboviruses identified in the study (MG22, 31, 36 and 49) with related viruses. Similarity rates are given in percent. (XLS 28 kb)

Additional file 3: Figure S1. The maximum likelihood analysis of the partial putative nucleocapsid protein (152 amino acids) of phleboviruses. The sequence characterized in this study is given in bold and indicated with the GenBank accession number, pool code and a black circle. Global virus strains are indicated by GenBank accession numbers, virus and strain/isolate names. Bootstrap values higher than 60 are shown. (PDF 422 kb)

Additional file 4: Table S3. Pairwise comparison of the nucleotide and putative amino acid sequences of the S segment of tick phleboviruses identified in the study (MG22) with related viruses. Similarity rates are given in percent. (XLS 25 kb)

Additional file 5: Table S4. Pairwise comparison of the nucleotide and putative amino acid sequences of the L segment of the Crimean-Congo hemorrhagic fever virus (CCHFV) identified in the study (MG48) with distinct CCHFV strains. Similarity rates are given in percent. (XLS 26 kb)

Additional file 6: Table S5. Pairwise comparison of the nucleotide and putative amino acid sequences of the M segment of the Crimean-Congo hemorrhagic fever virus (CCHFV) identified in the study (MG48) with distinct CCHFV strains. Similarity rates are given in percent. (XLS 26 kb)

Additional file 7: Table S6. Pairwise comparison of the nucleotide and putative amino acid sequences of the S segment of the Crimean-Congo hemorrhagic fever virus (CCHFV) identified in the study (MG48) with distinct CCHFV strains. Similarity rates are given in percent. (XLS 26 kb)

## Abbreviations

CCHFV: Crimean-Congo hemorrhagic fever virus
DENV: Dengue virus
JEV: Japanese encephalitis virus
L: Large (genomic segment)
M: Medium (genomic segment)
NGS: Next generation sequencing
ORF: Open reading frame
RT: Reverse transcription
S: Small (genomic segment)
SFTSV: Severe fever with thrombocytopenia syndrome virus;
TBEV: Tick-borne encephalitis virus
WNV: West Nile virus
YFV: Yellow fever virus
ZIKV: Zika virus
