# Supplementary material for: Generic amplification and next generation sequencing reveal Crimean-Congo hemorrhagic fever virus AP92-like strain and distinct tick phleboviruses in Anatolia, Turkey
Source: Parasit Vectors. 2017 Jul 14;10:335. doi: 10.1186/s13071-017-2279-1 (PMC5513282; doi:10.1186/s13071-017-2279-1)
Supplement: Supplementary file 3 — The maximum likelihood analysis of the partial putative nucleocapsid protein (152 amino acids) of phleboviruses. The sequence characterized in this study is given in bold and indicated with the GenBank accession number, pool code and a black circle. Global virus strains are indicated by GenBank accession numbers, virus and strain/isolate names. Bootstrap values higher than 60 are shown. (PDF 422 kb) [file 13071_2017_2279_MOESM3_ESM.pdf]

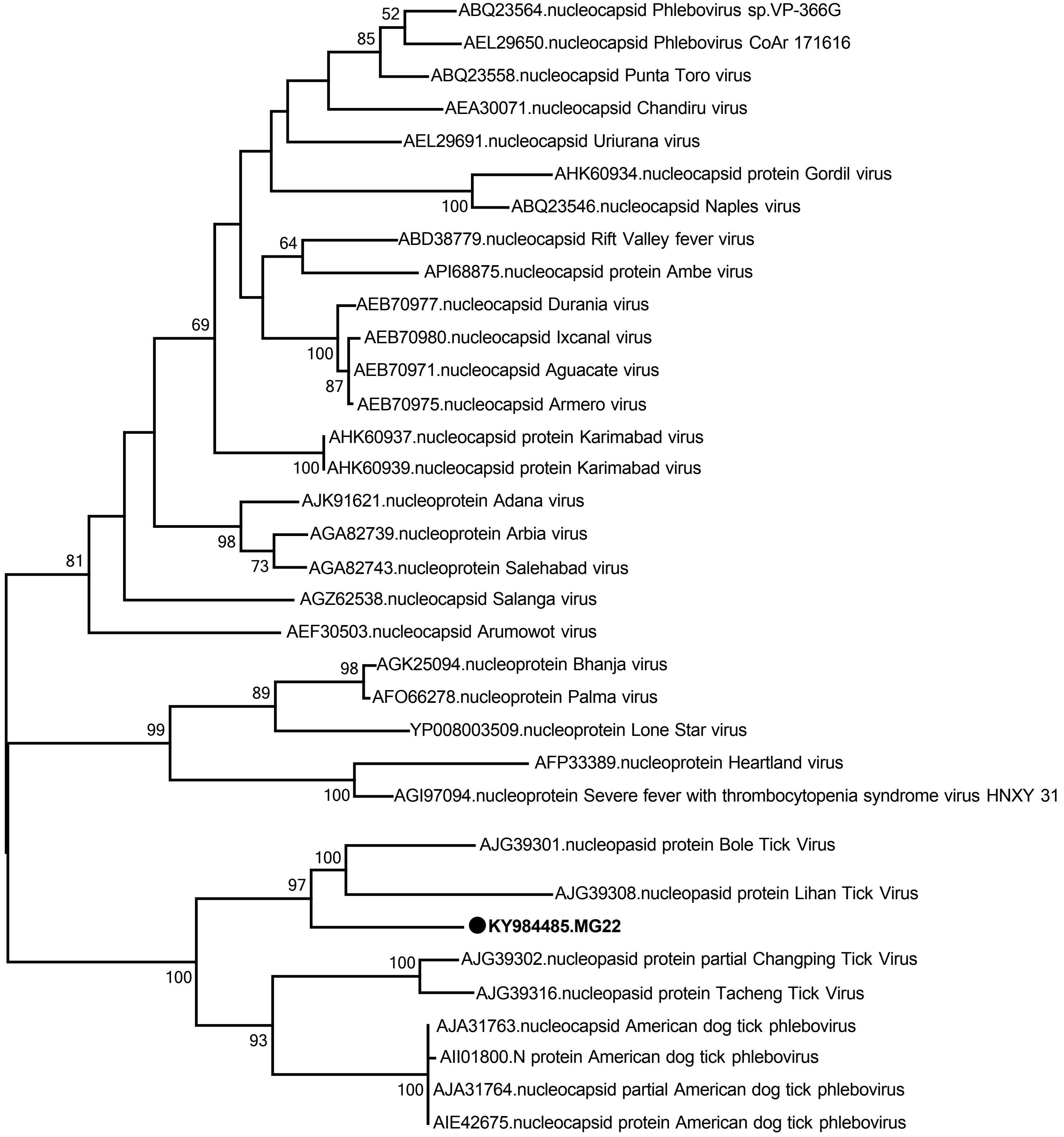

Additional file 3: Figure S1. The maximum likelihood analysis of the partial putative nucleocapsid protein (152 amino acids) of phleboviruses. The sequence characterized in this study is given in bold and indicated with the GenBank accession number, pool code and a black circle. Global virus strains are indicated by GenBank accession numbers, virus and strain/isolate names. Bootstrap values higher than 60 are provided.
